# *Fangumellus flavobadius*: a new genus and species of plant bug from Laos (Heteroptera, Miridae, Mirinae, Mirini)

**DOI:** 10.3897/zookeys.603.9063

**Published:** 2016-07-06

**Authors:** Tomohide Yasunaga, Minsuk Oh, Seunghwan Lee

**Affiliations:** 1 Research Associate, Division of Invertebrate Zoology, American Museum of Natural History, New York c/o Nameshi 2-33-2, Nagasaki 852-8061, Japan; 2 Biosystematics Laboratory, Research Institute for Agriculture and Life Sciences, Seoul National University, Seoul 151-921, Republic of Korea; 3 Department of Agricultural Biotechnology, Seoul National University, Seoul 151-921, Republic of Korea

**Keywords:** Miridae, Mirinae, Mirini, new genus, new species, Fangumellus flavobadius, Laos, taxonomy

## Abstract

A new species of the plant bug tribe Mirini representing a new genus, *Fangumellus flavobadius*, is described from Laos. This genus is characterized primarily by the medium-sized, ovoid, tumid body, less shiny, roughened, almost impunctate dorsal surface, short antenna and labium, short pygophore, and atypical shape of parameres and endosoma. The phylogenetic relationship to other known mirine genera is also discussed.

## Introduction

Fauna of the plant bug family Miridae in the Lao People’s Democratic Republic is still in great need of investigation. This paper represents a part of recent attempt to document the plant bug fauna of Laos, subsequent to Oh et al. (2015) and Yasunaga and Duwal (2015).

The present work documents an undescribed species of the plant bug family Miridae, which cannot be placed in any known genera. This bug, belonging to the tribe Mirini of the subfamily Mirinae, has several atypical features, in particular the shape of the pygophore and parameres, although its conventional ovoid body form is reminiscent of some taxa of *Lygus*-complex. Among nearly 300 described genera in the Mirini, approximately 40 genera may be assigned to this complex group in Asia (Schwartz and Footitt 1998, Yasunaga et al. 2002). We herein describe a new genus *Fangumellus* to accommodate this peculiar new mirid species, *Fangumellus flavobadius*, and discuss its phylogenetic position.

## Materials and methods

The holotype is deposited in Biosystematics Laboratory, Research Institute for Agriculture and Life Sciences, Seoul National University, Seoul, Korea (SNUK). Matrix code label is attached to the holotype, which uniquely identifies each specimen and is referred to as ‘unique specimen identifiers’ (USIs). The USI code [AMNH_PBI 0123] comprises a dataset code (AMNH_PBI) and a unique specimen number (0123). These data were digitized on the Arthropod Easy Capture (formerly the Planetary Biodiversity Inventory) database maintained by the American Museum of Natural History, New York, USA (http://research.amnh.org/pbi/) and are incorporated with http://www.discoverlife.org.

All measurements are in millimeters. Terminology of the male genitalia follows Schwartz and Foottit (1998) and Yasunaga and Schwartz (2007). Further information on known taxa mentioned in the text is available on website (Schuh 2002–2014). Digital images used in this paper were captured using a Diagnostic Instruments Insight Camera 14.2 Color Mosaic, with a SPOT Insight System. The following abbreviations are used for the male genitalia (Fig. 2): GP, secondary gonopore; HP, hypophysis; PT, phallotheca; SD, seminal duct; SL, sensory lobe; SP, spiculum.

## Results

### Fangumellus gen. n.

Taxon classificationAnimaliaHemipteraMiridae

http://zoobank.org/09B11573-23B5-4AEB-A0D1-6A6B437FCAB7

#### Type species.

*Fangumellus flavobadius* sp. n.

#### Diagnosis.

Distinguished from other genera in tribe Mirini by the following combination of characters: Medium-sized, ovoid, tumid body; less shining, partly matte, almost impunctate dorsal surface; short antenna and labium; short pygophore; and unique shape of parameres and endosoma (Fig. 2), especially sinuate distal portion of right paramere.

#### Description.

**Figure 1.**
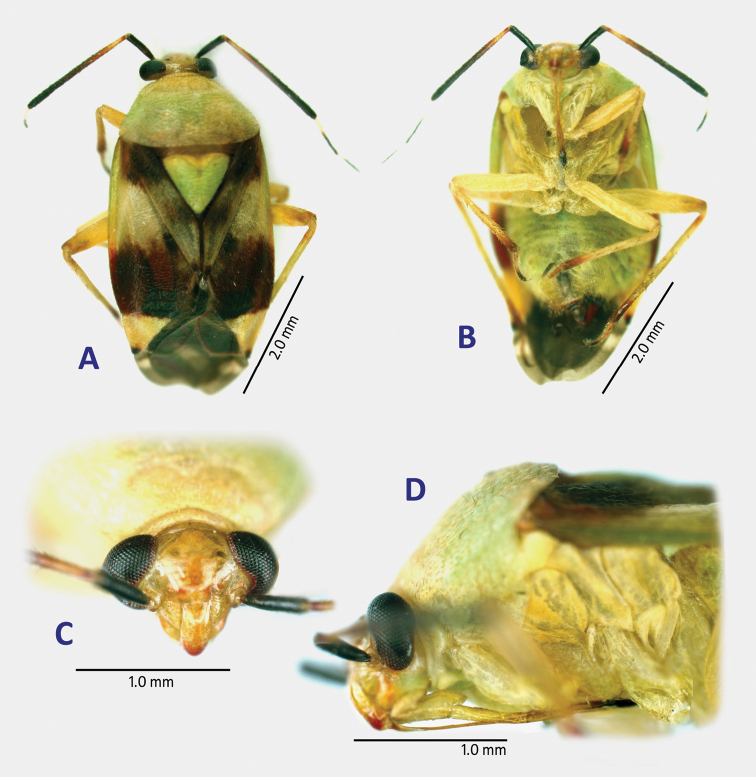
Habitus images of *Fangumellus flavobadius*, holotype male. **A** dorsal view **B** ventral view **C** head in frontal view **D** head and thorax in left lateral view.

**Figure 2.**
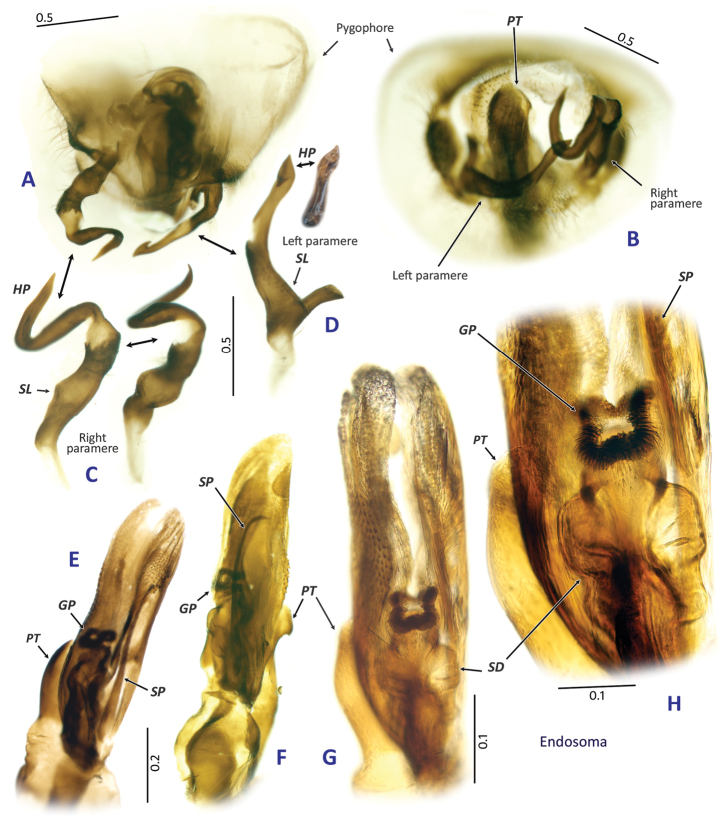
Male genitalia of *Fangumellus flavobadius*. **A** pygophore in ventral view **B** pygophore in caudal view **C** right paramere **D** left paramere **E−H** endosoma. Abbreviations corresponding to those mentioned in materials and methods section.

***Male***: Body medium-sized, ovoid, tumid (Fig. 1A); dorsal surface weakly shining, with uniformly distributed, pale brown, short, reclining setae. **Head**: Vertical, smooth; eye rather small; vertex weakly carinate basally; frons neither serrate nor sulcate; clypeus weakly swollen (Fig. 1C). **Antenna**: Generally short, not thickened or clavate, lacking noticeable long setae or spines; segment I subequal in length to IV; segment II almost linear, about as thick as I, shorter than basal width of pronotum; segments III and IV filiform. **Labium**: Short, slender, reaching subapical part of mesocoxa (Fig. 1B). **Thorax**: Pronotum shagreened or matte, shallowly and irregularly punctate, with narrow calli, not carinate laterally; collar somewhat arched, about as thick as base of antennal segment II; scutellum weakly shining, rather tumid, shallowly and transversely wrinkled; pleura weakly shagreened or matte; metathoracic scent efferent system as in Fig. 1D. **Hemelytron**: Less shining, weakly shagreened, with uniformly distributed, whitish, silky, reclining setae. **Legs**: Generally short; tibial spines dark, short, sparsely distributed; meta-tarsomere I subequal in length to II; meta-tarsomere III longer than I or II. **Genitalia** (Fig. 2): Pygophore short, with triangular apex (Fig. 2A). Parameres quite atypical in shape, generally slender and elongate (Fig. 2A−D); left paramere with hooked apex of hypophysis and a thumblike, blunt-tipped protuberance on sensory lobe (Fig. 2D); right paramere sigmoid, with somewhat spiral or coiled hypophysis (Fig. 2C). Endosoma as in Fig. 2A−D, with a slender, apically hooked spiculum; secondary gonopore thick-rimmed, without any accompanied sclerite; seminal duct well expanded subapically (Fig. 2H); phallotheca slender, with a folded apex (Fig. 2F). ***Female***: Unknown.

#### Etymology.

Named after the King ‘Fa Ngum’ who first established a unified kingdom (Lan Xang Kingdom) in Laos in 14th century, combined with Latin diminutive (-ellus); masculine.

#### Discussion.

This new genus is at first sight reminiscent of *Pachylygus* Yasunaga or some taxa of *Lygus* (in broad sense, see Schwartz and Foottit 1998, Yasunaga et al. 2002). However, the less punctate and rather shagreened dorsal surface and atypical shape of the parameres suggest that *Fangumellus* is evidently not closely related to those taxa. It is our opinion that comparison with *Paramiridius* Miyamoto & Yasunaga may merit careful consideration. One of *Paramiridius* species recently described from Laos, *Paramiridius laomontanus* Oh, Yasunaga & Lee, has some similarities in general appearance and male genitalia (e.g., ovoid body, impunctate dorsum, short labium, slender and apically hooked left paramere, thick-rimmed secondary gonopore, and apically expanded seminal duct) (Oh et al. 2015). Nonetheless, some of these similarities appear homoplasious or are shared by other mirine taxa. We currently cannot determine any sister taxon closely related to *Fangumellus*; a broader survey including the female genitalic structure is required to demonstrate its closest relative.

We can only suggest herein that the two unique characters exhibited on the parameres are in all likelihood autapomorphies for the new taxon (sigmoid, spiral, elongate hypophysis of right paramere and a thumb-like, subbasal protuberance of left paramere, which are not possessed by any other known mirine genera). In addition, the surface structure of *Fangumellus* (e.g., shagreened, impunctate dorsum with rather stiff vestiture) may be presumed as a derived character.

### Fangumellus flavobadius sp. n.

Taxon classificationAnimaliaHemipteraMiridae

http://zoobank.org/31A8801B-B1B5-45CA-96BB-3E1C9E1AF025

Figs 1 , 2

#### Type material.

Holotype male. LAOS: Xiang Khoang Prov., Kham Dist., Phosabous National Protected Area, Namchack Village, [N19°50'57", E103°47'51", 670m alt.], light trap, 2 May 2015, Oh (Coll. No: 150429-MS-29) (AMNH_PBI 00380463).

#### Diagnosis.

Recognized by the characters mentioned in generic diagnosis and distinctive color pattern. Most similar in general appearance to certain species of *Lygus* Hahn, *Pachylygus* Yasunaga or *Peltidolygus* Poppius (cf. Schwartz and Foottit 1998, Yasunaga et al. 2002); distinguished by somewhat shagreened pronotum without clear punctures, rather flat, not developed scutellum and unique shape of parameres.

#### Description.

***Male***: Body yellow, partly tinged with olive green (yellow parts assumed to be more or less greenish when alive); dorsal surface weakly shining, rather matte or roughened, with reddish brown pattern on hemelytron (Fig. 1A). **Head**: Pale brown, shining (Fig. 1C); apex of clypeus narrowly rouge (Fig. 1D). **Antenna**: Dark brown; basal quarter of segment II pale reddish brown; basal 1/3 parts of segments II and III creamy yellow. Labium shiny pale brown; apical half of segment IV darkened (Fig. 1B, D). **Thorax**: Pronotum yellowish brown, weakly wrinkled and faintly punctate, with pale olive disk; calli and collar yellowish brown; mesoscutum pale brown; scutellum olive green, shallowly wrinkled; pleura including scent efferent system yellowish brown; propleuron faintly punctate as in disk (Fig. 1D). **Hemelytron**: Pale brown, weakly shining, with two reddish brown, noticeable maculae at base of corium across base of clavus and at posterior half of corium to embolium (Fig. 1A); clavus with an obscure mark at middle and narrowly reddish brown apex; cuneus yellowish brown, with darkened apex; membrane smoky brown, with an yellow spot posterior to apex of cuneus. **Legs**: Coxae and legs yellowish brown (Fig. 1B); each coxa and trochanter slightly tinged with olive; apex of metafemur slightly darkened; apices of all tibia reddish brown; all tarsi pale reddish brown; each tarsomere III dark brown. **Abdomen**: Yellow, widely tinged with green; median parts of abdominal tergites sanguineous. Male genitalia as mentioned in generic description. ***Female***: Unknown.

#### Measurements

(in mm). Holotype male: Total body length 5.72; head width including eyes 1.18; head height 0.82; vertex width 0.46; lengths of antennal segments I−IV 0.56, 1.80, 0.73, 0.55; total length of labium 1.56; mesal pronotal length 1.18; basal pronotal width 2.21; maximum width across hemelytron 2.63; lengths of metafemur, tibia and tarsus 1.80, 2.57, 0.62; and lengths of meta-tarsomeres I−III 0.21, 0.22, 0.35.

#### Etymology.

From Latin, flavus (= yellow) combined with badius (= maroon or chestnut brown), referring to the basic color pattern of this new species; an adjective.

#### Distribution.

Laos (Xiang Khoang).

#### Biology.

Unknown; only one male was collected using UV light trap.

#### Discussion.

This new species evidently represents a member of *Lygus* sensu lato. In the key to species of this complex group from Indo-Australian region (Poppius 1914), *Fangumellus flavobadius* actually keys out to *Lygus* [s.l.] *dohrni* Poppius, 1914, described from Sumatra, Indonesia. However, this mirid is distinct in having the following characters: Body elongate and large (6.5 mm in total length, 2.5 mm maximum width); apex of clypeus; dark membrane with yellow veins; antennal segment II 2.5 times as long as segment I; scutellum flat; clavus and corium rather strongly punctate than pronotum; and tibiae with brown spines, each of which has a dark, small dot. Judging from the original description by Poppius (1914), his taxon is more probably close to *Castanopsides* Yasunaga-*Mahania* Poppius group (cf. Yasunaga and Duwal 2006). Although several recent works (e.g., Schwartz and Chérot 2005) carefully revised the generic placements for the species assigned to the *Lygus*-complex, dozens of species are still placed in *Lygus* sensu lato, and, needless to say, require further critical revisions.

## Supplementary Material

XML Treatment for Fangumellus

XML Treatment for Fangumellus flavobadius
